# Comparison of Ciaglia and Griggs Percutaneous Tracheostomy Techniques – A Biomechanical Animal Study

**DOI:** 10.5005/jp-journals-10071-23174

**Published:** 2019-06

**Authors:** Ohad Ronen, Israel Rosin, Uri Zeev Taitelman, Edward Altman

**Affiliations:** 1 Department of Otolaryngology, Head and Neck Surgery, Galilee Medical Center, Bar Ilan University, Safed, Israel.; 2 Department Thoracic Surgery, Rambam Health Campus, Haifa, Israel.; 3 General Intensive Care Unit, Rambam Health Campus, Technion-Israel Institute of Technology, Haifa, Israel; 4 Department of Thoracic Surgery Unit, Galilee Medical Center, Bar Ilan University, Safed, Israel

**Keywords:** Animal model, Biomechanics, Percutaneous tracheostomy

## Abstract

**Background and aims:**

The two most common commercial percutaneous dilation tracheotomy (PDT) sets apply different techniques. Our aim was to investigate the biomechanical properties of these two techniques on an animal model, that simulate a human trachea.

**Materials and methods:**

Biomechanical properties of the different steps of the Ciaglia Blue Rhino® and Griggs Portex® techniques were measured on 20 pig cadavers.

**Results:**

We found that the use of the two different devices created equal sized openings in the trachea (*p* >0.05). The force needed to insert the Griggs forceps was 1.8 kg average compared to 2.51 kg using the Ciaglia dilator (*p* <0.00001). The calculated total energy expenditure in the Ciaglia Blue Rhino® kit was 1.46 times greater than the Griggs Portex® kit (*p* <0.0001). This was mainly due to the amount of energy required during the final dilator stage, which was 4 times more using the Ciaglia Blue Rhino® dilator than the Portex® Griggs-dilator forceps.

**Conclusion:**

We conducted a series of biomechanical properties experiments on an animal model of PDT using two popular commercial kits – Griggs Portex® guidewire dilating forceps by Smiths Medical and Ciaglia Blue-Rhino® by Cook Medical. The Ciaglia technique required almost 50% more energy to perform a PDT (*p* <0.0001), mainly because of the force exerted during the final dilator insertion stage compared to the Griggs forceps. Further research is needed to examine if these properties are related to some of the PDT complications.

**How to cite this article:**

Ronen O, Rosin I, Taitelman UZ, Altman E. Comparison of Ciaglia and Griggs Percutaneous Tracheostomy Techniques – A Biomechanical Animal Study. Indian J Crit Care Med 2019;23(6):247–250.

## INTRODUCTION

Two common approaches for percutaneous dilation tracheostomy (PDT) were described by Ciaglia in 1985, using a nephrectomy set,^1^ and Griggs in 1990 using a modified Kelly forceps with an inner channel for a guide wire.^2^ These two techniques are manufactured as commercial kits: Ciaglia Blue Rhino® (Cook Medical, Bloomington, Indiana) and Griggs Portex® (Smiths Medical, Minneapolis, Minnesota).

There are several articles comparing these two common techniques.^3–7^ first described in the 1950s, has become a common bedside technique in the Intensive Care Unit (ICU). While the authors compare complications rates, technical difficulty, and surgical duration of both techniques, these studies do not allow us to define the inherent properties causing these differences.

Our aim was, therefore, to compare the biomechanical properties of these two commercial kits for percutaneous tracheotomy on an animal model, that simulate a human trachea. We assumed that there are differences in the biomechanical properties during the different phases of both techniques. Such differences will allow us to choose the appropriate method for different patients.

## MATERIALS AND METHODS

The trial has been approved by the Institutional Review Board (NHR0097417 on June 2017). Animal experimentation committee approval was waived. Porcine necks were collected as “by products” of routine pig processing for human consumption; no pigs were killed for the purpose of this study.

We used an animal model of male pigs, with an average weight of 35–30 kg and an age range of 3.5–4 months. The male pig's trachea has the same dimensions as that of a human, and is used in other tracheal models.^8^

**Fig. 1:**
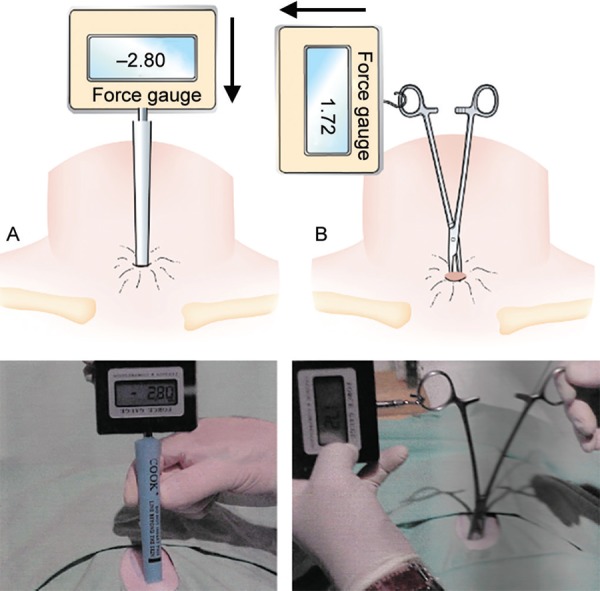
Measurements of the force used in each step of the procedures. The upper panel presents an illustration of the images below it. Panel A demonstrates measurement of a pushing force, and panel B demonstrates measurements of a traction force. Illustrations are courtesy of Dr. Amiel A. Dror

***Description of the animal model:*** The pig cadaver was placed on its back and the following anatomical landmarks were palpated and marked: thyroid cartilage, cricoid cartilage, and jugular notch. A 1.5 cm horizontal incision was made 1–1.5 cm inferior to the cricoid cartilage. Forceps were used for blunt dissection up to the trachea. Finger palpation was used to locate the 2nd and 3rd tracheal rings. The trachea was punctured in this location using a designated needle from the kit and the force used to puncture the trachea was measured using a dynamometer (FG5000A, MRC labs, Holon, Israel), as can be seen in Figure 1. The rest of the procedure was done according to the instructions supplied in the different kits.^9,10^ The amount of force required to insert the different dilators and the tracheotomy tube was measured using the dynamometer (Fig. 1). In all pigs, a tracheotomy tube number 8 was used (Portex® by Smiths Medical, Minneapolis, Minnesota). After the tracheotomy, tubes were inserted in the proper location, the tracheas and larynges were resected and the posterior membranous wall of the tracheas were dissected off. Pictures were taken of the inner part of the tracheal opening in both groups. The surgeon (EA) was blinded to the readings of the dynamometer that were collected by a different author (IR).

The following parameters were collected for each of the techniques used:

1. The force required to puncture the trachea by the supplied needle.
2. The force required to perform the first dilation by the supplied 14F dilator.
3. Width of the tracheal stoma made by the 14F dilator.
4. The force applied to the tracheal rings by inserting the last supplied dilator (Ciaglia) or forceps (Griggs).
5. The size and shape of the tracheal stoma after the insertion of the tracheotomy tube.
6. The force applied to insert a number 8.0 Portex® tracheostomy tube.

***Statistical analysis:*** Quantitative data was expressed as mean ± standard deviation, median, and range. Qualitative data was expressed as frequencies and percentages. We compared quantitative difference between the groups by an independent t-test or a Wilcoxon rank sum test as appropriate; the qualitative differences were compared with a Chi-square test or Fisher's exact test, as appropriate. The data were summarized using Microsoft Excel and then processed statistically using SPSS statistical package (Version 19). A *p* value less than 0.05 was considered statistically significant.

## RESULTS

Overall, ten surgeries were performed for each technique.

***Needle insertion:*** In both techniques the force needed to penetrate the trachea using the supplied needle when it was inserted through a tracheal ring cartilage was twice that when inserted between rings. We included only punctures that went between the rings. The Portex® needle pierced the trachea using a lighter force of 0.31 kg on average compared with 0.58 kg on average using the Cook Medical needle (*p* <0.00001).

***14F dilator:*** The Portex® 14F dilator needed an average force of 1.91 kg compared with 1.73 kg using the dilator in the Ciaglia Blue Rhino® kit. Both dilators created equal round shaped holes with sharp edges in the trachea. The opening sizes in the tracheal adventitia was 1.59 cm in the Portex® group vs 1.49 cm in the Ciaglia Blue Rhino® group and the mucosal opening size at this stage was 1.70 cm in the Portex® group vs 1.60 cm in the Ciaglia Blue Rhino® group, both with no statistical difference (*p* >0.05).

***The final dilator:*** The force exerted on the Portex® guidewire dilating forceps was on average 1.8 kg creating a 1.5 cm opening on average. This opening had a typical two-tears shape composed of a horizontal component of 1.7 cm average width (range 1.5–1.9 cm) and a longitudinal component of 1.6 cm average height (range 1.4–1.8 cm). The horizontal tear was directed in between the tracheal rings and the longitudinal tear involved only the mucosal layer. The Ciaglia Blue Rhino® dilator caused a typical tear that also had two components in which its horizontal tear was in between the tracheal rings and had an average width of 1.6 cm (range 1.5–1.8 cm). The force needed to insert the Ciaglia Blue Rhino® dilator was statistically different compared to opening by the Griggs forceps (2.51 kg vs 1.8 kg respectively, *p* < 0.00001).

***Insertion of the tracheotomy tube:*** The 8.0 tube was inserted using a 2.76 kg force on an average (range 2.35–2.95 kg) using the Portex® technique. This resembled the force needed to insert the tracheotomy tube using the Ciaglia Blue Rhino® technique in which an average 2.51 kg (range 2.1–2.8 kg) was needed (*p* > 0.05).

***Total work required:*** The calculated total energy expenditure using the Ciaglia Blue Rhino® technique was 62 kg*cm, almost 1.5 times greater than the Portex® technique (43 kg*cm), a difference that was found to be statistically significant (*p* < 0.0001). This was mainly due to the amount of energy required during the final dilator stage, which was 4 times more using the Ciaglia Blue-Rhino® dilator than the Portex® Griggs-dilator forceps.

**Fig. 2:**
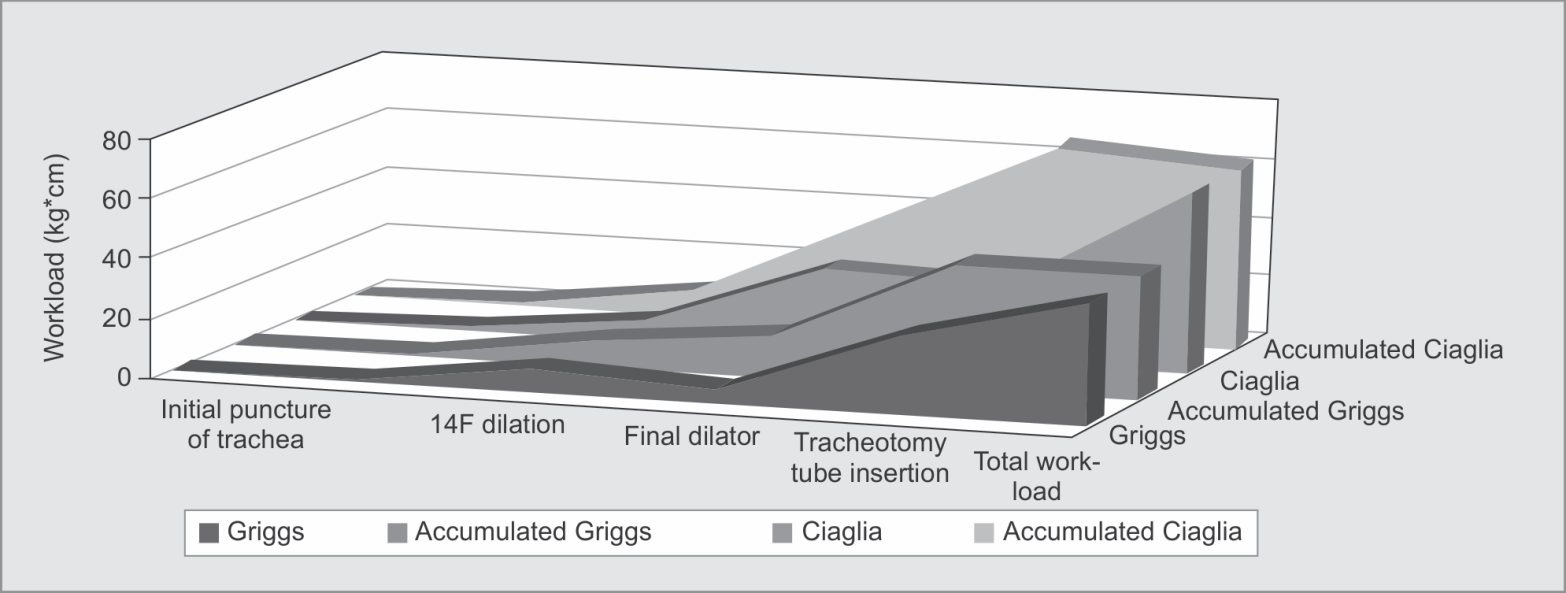
Breakdown of the energy exerted for each stage and the accumulated energy after each stage of percutaneous tracheotomy. Notice the energy difference during the insertion of the final dilator, which causes the final energy difference between the two kits examined. Blue – Griggs method, orange – accumulated energy using Griggs method, grey – Ciaglia method, yellow – accumulated energy using the Ciaglia method. Units are in kilogram-centimeter (kg*cm).

The above results are summarized in Tables 1 and 2, and Figure 2.

## DISCUSSION

In this animal model experiment, we compared the differences in biomechanical properties of two common PDT techniques. We found statistically significant differences in favor of the Griggs techniques such as lesser force during the piercing step, and the final dilator step which accumulated into lower workload during these steps. Consequently, the overall workload was significantly lower in the Griggs technique. A possible explanation is that the Griggs forcep's vector of force is parallel to the rigid cartilaginous tracheal rings while the vector of forces created by the Ciaglia dilatation is in all directions, including the rings. A study that prospectively compared the two techniques by endoscopically inspecting the trachea after the procedures found that tracheal stoma over-dilation was associated with the Griggs technique and rupture of tracheal rings was associated with the Ciaglia technique as might be explained by our findings.^11^

**Table 1:** The force and puncture size needed for each step of the two techniques

*Procedure step*	*Griggs Portex®* *(n = 10)*	*Ciaglia Blue Rhino®* *(n = 10)*	*p value*
Piercing force (kg)	0.31±0.05	0.58±0.10	<0.00001
14F Dilator insertion	1.91±0.23	1.73±0.17	0.059
Initial 14F opening size (cm)	0.51±0.03	0.52±0.05	0.597
Final dilator tearing force (kg)	1.80±0.19	2.51±0.21	<0.00001
Final adventitial opening size (cm)	1.70±0.14	1.60±0.21	0.077
Final mucosal opening size (cm)	1.59±0.11	1.49±0.07	0.034
Tube insertion force (kg)	2.76±0.18	2.65±0.23	0.264

Both the needle piercing force and the tearing force of the dilator needed less force using the Griggs Portex® technique; Results are presented as mean±standard deviations; kg, kilograms; cm, centimeters

**Table 2:** Workload needed in each technique

*Procedure step*	*Griggs Portex®* *(n = 10)*	*Ciaglia Blue Rhino®* *(n = 10)*	*p value*
Initial puncture of trachea (kg*cm)	0.92±0.15	1.74±0.29	<0.00001
14F dilation (kg*cm)	8.60±1.02	7.78±0.77	0.059
Final dilator (kg*cm)	5.41±0.57	25.81±2.46	<0.00001
Tracheotomy tube insertion (kg*cm)	27.58±1.76	26.94±2.28	0.264
Total workload (kg*cm)	42.51±1.46	62.27±8.01	<0.00001

The Griggs technique needed less energy than the Ciaglia technique mainly due to the final dilator stage; Results are presented as mean±standard deviations; F, french gauge; kg, kilograms; cm, centimeters

In the Griggs technique, dilatation of the trachea is achieved by passing dilating forceps over the guidewire. Opening these forceps forcibly dilates the tracheal aperture as well as any intervening tissue. Because the desirable tracheostomy site, inferior to the first or second tracheal rings, often corresponds to the anatomical location of the thyroid isthmus, the latter may be torn on opening the dilating forceps, thus increasing the risk of bleeding.^5^ By contrast, the Ciaglia serial dilators tamponade bleeding as they progressively dilate and proved extremely safe, even in the context of coagulopathy.^12^

The lack of marks on the Griggs dilating forceps requires experience and dexterity from the surgeon in order to avoid creating a too large opening or hemorrhage from the thyroid tissue. In comparison, special signs on the Ciaglia dilator enable creation of a precise opening in the trachea when inserting a tracheotomy tube. The final insertion of the tracheal tube using the Ciaglia rigid introducer with 40% more energy, as was shown in our study, might create a false tract or damage to the posterior wall of the trachea. A possible modification of PDT combining the two techniques, using the Griggs forceps just before the Ciaglia dilator with its special depth/width marks and its plastic reinforcing guiding catheter might reduce complications even more.

Although there is less force exerted in the Griggs technique, there is a potential for over-zealous insertion of the forceps through the posterior tracheal wall and even into the esophagus, particularly because the procedure is blind,^4^ and especially since unlike Ciaglia kit, the Griggs kit includes a guidewire without a plastic reinforcing guiding catheter that can leave the wire more prone to kinking and an associated risk of paratracheal tube placement during the procedure.

An early paper that compared the two techniques found equal operative time needed to insert the tracheotomy tube,^3^ while others^5^ have found the Ciaglia method longer to complete, probably due to the use of multiple dilators,^4^ thus causing more hypercapnia as well as minor bleeding, transient hypoxemia, and damage to posterior tracheal wall without emphysema. In a later study, there was no difference in operative time using the Ciaglia single dilator kit.^7,13^ No differences were found in major complications such as tension pneumothorax, posterior tracheal wall injury with subcutaneous emphysema, loss of airway with hypoxemia, loss of stoma with impossible re-catheterization, and conversions to another technique,^6^ while others found more complications in the Ciaglia technique.^5^

Some of the above complications described above might be explained by the biomechanical differences between the two techniques, although our set of experiments was not designed to do so. Further measurements during PDT procedures on humans is needed to reach conclusions out of our findings on an animal model.

## CONCLUSION

We conducted a series of biomechanical properties experiments on an animal model of PDT using two popular commercial kits – Griggs Portex® guidewire dilating forceps by Smiths Medical and Ciaglia Blue-Rhino® by Cook Medical. The force needed and energy utilized to perform each technique were measured and calculated. We found that the use of the two different devices created equal sized openings in the trachea (*p* > 0.05). However, the Ciaglia technique required almost 50% more energy to perform a PDT (*p* < 0.0001), mainly because of the force exerted during the final dilator insertion stage compared to the Griggs forceps. Further research is needed to examine if these properties are linked to some of the complications related to PDT.
